# The role of cognitive reserve on terminal decline: a cross‐cohort analysis from two European studies: OCTO‐Twin, Sweden, and Newcastle 85+, UK

**DOI:** 10.1002/gps.4366

**Published:** 2015-10-15

**Authors:** Dorina Cadar, Blossom C. M. Stephan, Carol Jagger, Boo Johansson, Scott M. Hofer, Andrea M. Piccinin, Graciela Muniz‐Terrera

**Affiliations:** ^1^ MRC Unit for Lifelong Health and Ageing at University College LondonLondonUK; ^2^ Institute of Health and SocietyNewcastle UniversityNewcastleUK; ^3^ Department of PsychologyUniversity of GothenburgGothenburgSweden; ^4^ Department of PsychologyUniversity of VictoriaVictoriaCanada

**Keywords:** cognition, death, dementia, MMSE, education, terminal decline, cognitive reserve, longitudinal study, mixed multilevel

## Abstract

**Objective:**

Cognitive performance shows a marked deterioration in close proximity to death, as postulated by the terminal decline hypothesis. The effect of education on the rate of terminal decline in the oldest people (i.e. persons 85+ years) has been controversial and not entirely understood. In the current study, we investigated the rate of decline prior to death with a special focus on the role of education and socioeconomic position, in two European longitudinal studies of ageing: the Origins of Variance in the Old‐Old: Octogenarian Twins (OCTO‐Twin) and the Newcastle 85+ study.

**Methods:**

A process‐based approach was used in which individuals' cognitive scores were aligned according to distance to death. In a coordinated analysis, multilevel models were employed to examine associations between different markers of cognitive reserve (education and socioeconomic position) and terminal decline using the mini‐mental state examination (MMSE), controlling for age at baseline, sex, dementia incidence and time to death from the study entry to the time of death within each cohort.

**Results:**

The current findings suggest that education was positively associated with higher MMSE scores prior to death in the OCTO‐Twin, but not in the Newcastle 85+ study, independent of socioeconomic position and other factors such as baseline age, sex and time to death from the study entry. However, education was not associated with the rate of terminal decline in both of these studies.

**Conclusions:**

Our results offer only partial support to the cognitive reserve hypothesis and cognitive performance prior to death. © 2015 The Authors *International Journal of Geriatric Psychiatry* Published by John Wiley & Sons Ltd.

## Introduction

Significant evidence suggests that cognitive performance shows a marked deterioration in close proximity of death, known as ‘terminal decline’ (e.g. Sliwinski *et al.,* 2006, Wilson *et al.,* 2007, Thorvaldsson *et al.,* 2008, MacDonald *et al.,* 2011, Muniz‐Terrera *et al.,* 2011, Piccinin *et al.,* 2011, Muniz‐Terrera *et al.,* 2013). This emphasises a within‐person process of change from a preterminal phase of relative stability into more rapid decline that ends with death (Kleemeier, 1962, Riegel and Riegel, 1972). However, this theoretical conceptualisation lacks specificity distinguishing those experiencing serious neurobiological compromise such as dementia from those who remain free of dementia before death. A better understanding of this differentiation could carry distinct theoretical and clinical implication (e.g. Anstey *et al.,* 2006, Laukka *et al.,* 2006).

From many key modifiable risk factors, education received substantial interest in the field of dementia research, but its predictability of terminal decline is not entirely understood. The association between education and terminal decline remains highly controversial. For example, there are some evidence suggesting that education may modify the association between levels of cognitive function in proximity to death and Alzheimer's pathology assessed post‐mortem (Bennett *et al.,* 2003), while other studies support the association between education and level of cognitive performance, but not the association with the rate of cognitive decline prior to death (Johansson *et al.,* 2004, Laukka *et al.,* 2006, MacDonald *et al.,* 2006, Piccinin *et al.,* 2011). Furthermore, education appears to delay the onset of terminal decline in the oldest people (Muniz‐Terrera *et al.,* 2014) and to impact differently the rate of terminal decline according to various cognitive domains (Batterham *et al.,* 2011).

Often used as a proxy for cognitive reserve, education has been shown to be protective against faster rates of cognitive decline from middle to later life, in healthy individuals (Richards and Sacker, 2003, Stern, 2002), and related to both a delayed onset of dementia (Stern, 2009, Stern *et al.,* 1999) and a delayed onset of terminal decline (Muniz‐Terrera *et al.,* 2014). However, from a life‐course perspective, neuropathological burden could be considered as either a potential mediating factor between intelligence and mortality or a prior cause, if a suboptimal neurodevelopment was the antecedent for both the neurological condition and early mortality (Batty *et al.*, 2007). In light of this possibility, the curvilinear age trends for cognitive function in late life could actually be an artefact of accumulation over time, in individuals with different neuropathological burden and cognitive symptomatology. The aggregate function could be influenced by the increasing risk of terminal decline and its curvature, reflecting an average terminal decline within persons who are still cognitively stable (Singer *et al.,* 2003). As a result, it is less clear, whether education, or other markers of cognitive reserve such as occupation, could moderate terminal decline in the oldest people, because the nature of this decline may or may not follow the assumptions of flexible and efficient use of pre‐existing cognitive reserve (Stern, 2002). In addition, it is unclear if cognitive reserve will differentially influence the rates of decline prior to death for individuals who remain non‐demented and those diagnosed with dementia.

The aim of our study was to examine rates of terminal decline in mini‐mental state examination (MMSE) and the role of education and socio‐economic position (SEP) on these trajectories, using a coordinated analysis of two European population‐based samples of the oldest people from Sweden and the UK. In addition, we explored these trajectories among healthy individuals and those who developed dementia during the study period. In this respect, this cross‐cohort investigation represents a unique evaluation of consistency in patterns of associations for education and SEP as suggested markers of cognitive reserve. We analysed performance prior to death and the rate of change from study entry to time of death, reducing potential sources of heterogeneity that could emerge from differences in the analytical methodologies employed.

## Methods

### Study population OCTO‐Twin, Sweden

The first sample used in these analyses was from the comprehensive longitudinal Origins of Variance in the Old‐Old: Octogenarian Twins (known as the OCTO‐Twin study) based on the oldest cohort of the Swedish Twin Registry. The sample includes 702 participants, with 351 complete twin pairs born in 1913 and earlier, who were, or became, 80 years of age during the first wave of data collection (1991–1993). Participants have been assessed on five occasions at two‐year intervals, for a total of up to eight years of follow‐up. The average rate of attrition from one testing wave to the next was 20% (10% per year) and was primarily because of death (Cederlof and Lorich, 1978, McClearn *et al.,* 1997, Pedersen *et al.,* 2002).

### Study population Newcastle 85+, UK

The second sample was drawn from the Newcastle 85+ study; a longitudinal population‐based study of health and ageing. The sampling frame comprised all people born in 1921, recruited at age 85 during 2006–2007 (phase 1) when recruitment commenced and was broadly representative of 85 years old in England and Wales in terms of gender, residence in a care home and living alone. Participants have been re‐tested on three occasions (phases 2, 3 and 4) at approximately 18‐month intervals; however, the cognitive data were not available at phase 2, because of time restrictions. All assessments were conducted by a trained research nurse in the participants' residence (for full details see Collerton *et al.* (2007) and Collerton *et al.* (2009)). Most of post‐baseline attrition was because of death and participants' withdrawal (Davies *et al.,* 2014).

### Ethics

The OCTO‐Twin study received approval from the Ethics Committee at the Karolinska Institute in Stockholm and from the Swedish Data Inspection Authority in Sweden. The Newcastle 85+ study received ethical approval from the North Tyneside 1 Research Ethics Committee in the UK. Informed written consent was obtained from all participants in each study, or their carer, where capacity to consent was questionable, in case of severe cognitive impairment or dementia.

### Cognitive assessments

In both studies, global cognitive function was assessed with the MMSE (Folstein *et al.,* 1975), one of the most commonly used screening instruments for cognitive impairment. The test measures various domains including the following: orientation, registration (immediate memory), short‐term memory and attention, the ability to follow verbal and written commands, writing and copying. The MMSE scores range from 0 to 30, with high values indicating healthier cognitive status. The MMSE was administered at each of the five waves in OCTO‐Twin and only at three waves in Newcastle 85+ (baseline, phase 3 and phase 4 at approximately 18‐month intervals).

### Dementia diagnosis

In both studies, dementia was diagnosed by consensus according to the revised third edition of the Diagnostic and Statistical Manual of Mental Disorders. In OCTO‐Twin, dementia prevalence was identified by a multi‐disciplinary team consisting of a physician, a research nurse and two neuropsychologists, who reviewed the cognitive test results and medical records at each new wave (van den Kommer *et al.,* 2009). In the Newcastle 85+, dementia prevalence was determined at each wave, based on a review of data from general practice records alone (Collerton *et al.,* 2009).

### Covariates

Educational attainment was recorded in each cohort as a continuous measure, defined as the number of years of education. Information related to SEP were obtained during the home interview or via questionnaires within each study and classified into three occupational categories (low, medium and high SEP). For more information see Supporting Information. These categories represent general and well‐differentiated employment occupations in modern societies (Chandola, 2000).

Other covariates included sex, baseline age, incident dementia and the distance to death from the study entry.

### Data analyses and analytic approach

We undertook a coordinated analysis across the two cohort studies, employing the same statistical methodology within each study, to allow comparisons between the results and to examine consistency of patterns in terminal decline in these studies. The analytical sub‐samples included only data from individuals who died during the study and who were free of dementia at study entry. By excluding dementia cases at the study entry, we examined and compared the rates of terminal decline between those who developed dementia during the study period and those who remained free of dementia over the study period within each study. We derived an indicator variable (0 = no dementia, 1 = diagnosed with dementia during study follow up time) to account for individuals with dementia, and we obtained distinct estimates within each study.

A series of mixed models were fitted to MMSE to estimate trajectories of decline in global cognitive performance prior to the time of death, by employing regular mixed models and Tobit models independently within each study. Tobit models are often used to model trajectories of outcome variables that have floor or ceiling effects, such as MMSE. Bayesian Information Criteria (BIC), an index that combines model parsimony and goodness of fit (Raftery 1986), was used for model selection, and based on this we selected to report the results from the Tobit models.

All models used maximum likelihood estimation. Missing observations were assumed to be missing at random (Little and Rubin, 2002), and model assumptions were verified by examining residuals computed from the predicted values. For more information see Supporting Information.

Cognitive change was modelled as a linear function of time to death in both datasets and as a quadratic function in the OCTO‐Twin study given the larger number of measurements available in the Swedish study. The linear slopes represent annual rate of change (i.e. increase and decrease) per year closer to death and when a quadratic model is fitted (in OCTO‐Twin study), the linear term represents rate of change at the intercept and the quadratic term represents change (i.e. increase or acceleration and decrease or deceleration) in rate of change.

Level and rate of change were adjusted for demographic characteristics including sex (male participants used as reference category), years of education (centred at the mean value of 7 (OCTO‐Twin) or 10 (Newcastle 85+) years), age at study entry (centred at 83 (OCTO‐Twin) or 85 (Newcastle 85+) years) and time to death from the study entry (centred at −6 years to death (OCTO‐Twin) or −2.6 years to death (Newcastle 85+)). Given this model specification, the intercept represents the level of cognitive performance at this centred distance from death, for an individual with values of zero on all covariates in each study investigated.

Although the age range at the study entry of the individuals in each study is relatively narrow (mean age = 83.5, SD = 3.0 in the OCTO‐Twin study and mean age = 85.4, SD = 0.44 in the Newcastle 85+), those who joined the study at the younger age are expected to perform differently than those who joined the study at older age. Similarly, the performance of individuals who entered the study at a closer time to death was expected to differ from those who entered the study at a time further from death. To account for these differences (between‐person differences in age at study entry and distance to death), we adjusted our models for distance to death and age at study entry (Piccinin *et al.*, 2011).

Data analyses were conducted using the following statistical programmes: Mplus (version 6.11), Computer Software Los Angeles, CA (Muthén and Muthén, 2007) for the Regular Mixed and Tobit regression models (see the Mplus script as Supporting Information); STATA (StataCorp, 2013) Stata Statistical Software: Release 13. College Station, Texas: StataCorp LP for descriptive analyses and R Studio (R Development Core Team, 2010) for figures.

## Results

Demographics and cognitive scores at baseline and each follow‐up wave for the two studies (OCTO‐Twin and Newcastle 85+) are presented in Tables 1 and 2 respectively. Participants diagnosed with dementia at baseline (98 in OCTO‐Twin; 66 in Newcastle 85+) were excluded from the current analyses.

**Table 1:** Characteristics of study participants in the OCTO‐Twin

OCTO‐Twin study waves of testing					
	Wave 1	Wave 2	Wave 3	Wave 4	Wave 5
	1991–1993	1993–1995	1995–1997	1997–1999	1999–2001
Study sample^a^	556	457	351	256	174
Cumulative dementia cases	98^b^	42	80	100	126
Age (Mean/SD)	83.5 (3.0)	85.4 (3.0)	87.2 (2.7)	89.1 (2.8)	90.9 (2.4)
Female (%)	63.5%	62.8%	63.8%	68.7%	72.4%
MMSE included sample (mean/SD)	26.4 (4.0)	25.3 (5.4)	24.4 (6.0)	23.1 (7.7)	21.6 (7.7)
MMSE non‐dementia cases (mean/SD)	26.7 (3.9)	26.4 (4.4)	26.7 (4.2)	25.7 (5.6)	23.9 (5.7)
MMSE dementia cases (Mean/SD)	25.2 (3.8)	22.2 (6.7)	18.6 (8.5)	16.9 (8.4)	13.7 (8.3)
Education range	0–23 years	0–20 years	0–20 years	0–18 years	0–18 years
(mean/SD)‐total included sample	7.2 (2.3)	7.2 (2.3)	7.2 (2.3)	7.1 (2.1)	7.1 (2.2)
Education range	0–23 years	0–20 years	0–20 years	0–18 years	0–18 years
(Mean/SD) non‐dementia cases	7.2 (2.3)	7.3 (2.4)	7.3 (2.4)	7.2 (2.2)	7.1 (2.2)
Education range	2–17 years	2–17 yrs	2–13 years	2–13 years	2–13 years
(Mean/SD) dementia cases	7.0 (1.8)	7.0 (1.8)	6.8 (1.6)	6.8 (1.7)	6.7 (1.8)
Average time to death (years) from each wave (mean/SD)	−6.0 (3.8)	−4.8 (3.4)	−3.9 (2.8)	−3.1 (2.2)	−2.1 (1.7)

a Sample of individuals who died during the study period in OCTO‐Twin, representing those included in the current analyses.

b Dementia cases at baseline (*N* = 98 in OCTO‐Twin study) have been excluded from these analyses.

**Table 2:** Characteristics of study participants in the Newcastle 85+

Newcastle 85+ study waves of testing			
	Wave 1	Wave 3^a^	Wave 4
	2006–2007	2009–2010	2011–2012
Study sample^b^	845	485	344
Cumulative dementia cases	66^c^	116	139
Age (Mean/SD)	85.4 (0.44)	88.4 (0.39)	90.5 (0.40)
Female (%)	55.6%	55.6%	53.7%
MMSE included sample (Mean/SD)	25.8 (5.3)	25.4 (5.4)	24.8 (6.3)
MMSE non‐dementia cases (mean/SD)	26.0 (4.6)	26.3 (4.2)	26.9 (3.7)
MMSE dementia cases (mean/SD)	15.3 (8.5)	15.6 (8.2)	13.1 (9.0)
Education range	6–20 years	7–19 years	9–15 years
(Mean/SD) total included sample	9.9 (1.8)	10.1 (1.9)	10.3 (1.6)
Education range	6–20 years	7–19 years	9–15 years
(Mean/SD) non‐dementia cases	9.9 (1.8)	10.2 (2.1)	10.4 (2.0)
Education range	8–17 years	8–17 years	9–12 years
(Mean/SD) dementia cases	9.9 (1.6)	9.8 (1.6)	10.3 (1.2)
Average time to death (years) from each wave (mean/SD)	−2.6 (1.5)	−1.4 (0.7)	−0.3 (0.2)

a MMSE was not administered at wave 2, so wave 3 was considered as time 2 and wave 4 was considered as time 3 in these analyses.

b Sample of individuals who died during the study period in Newcastle 85+, representing those included in the current analyses.

c Dementia cases at baseline (*N* = 66 in Newcastle 85+ study) have been excluded from these analyses.

The majority of participants in each study were women (64% in OCTO‐Twin; 56% in Newcastle 85+). The number of years of education for participants who remained free of dementia during the study period, in OCTO‐Twin, ranged from 0 to 23 years at baseline and from 0 to 18 years at the last follow‐up (wave 5). In contrast, for the incident dementia cases in this study, education ranged from 2 to 17 years at baseline and from to 2 to 13 years at the last follow‐up. In the Newcastle 85+ study, education ranged from 6 to 20 years at baseline and from 8 to 17 years at the last wave for non‐cases. For those with dementia in this study, education ranged from 8 to 17 years at baseline and from to 9 to 12 years at the last follow‐up.

Estimates and standard errors for the MMSE intercept and slope across the two cohorts are shown in Table 3. Longitudinal changes in MMSE scores were modelled as a linear function in both studies and also as a quadratic function in the OCTO‐Twin study. We used the lowest BIC scores to select the best model fit (BIC regular model = 10121.96 and BIC Tobit model = 9442.56 in OCTO‐Twin, BIC regular model = 2885.10 and BIC Tobit model = 2727.63 in Newcastle 85+ study); therefore, we reported the results from the Tobit models.

**Table 3:** Mean, standard error of the estimates of the effect of risk factors on random effects of terminal decline mixed model for MMSE within each study

	MMSE in OCTO‐Twin study			MMSE in Newcastle 85+ study		
	*Coef.*	*SE*	*p‐value*	*Coef.*	*SE*	*p‐value*
*Fixed effects*			*N* = 552			*N* = 352^a^
Intercept (Level of performance on MMSE prior to death)	22.44	0.68	<0.001	24.60	0.62	<0.001
Years to death from baseline	0.48	0.09	<0.001	0.10	0.23	0.67
Baseline age	−0.49	0.10	<0.001	−0.13	0.83	0.88
Education	0.44	0.14	0.009	0.19	0.23	0.44
Socio‐economic position Medium	0.60	0.78	0.73	0.80	1.15	0.49
High	0.88	1.09	0.44	1.43	0.92	0.12
Female	0.85	0.16	0.41	−0.48	0.79	0.55
Dementia cases	−12.13	0.90	<0.001	−10.68	1.48	<0.001
Linear slope (rate of decline)	−1.60	0.21	<0.001	−1.08	0.23	<0.001
Years to death from baseline	−0.02	0.05	0.61	−0.26	0.09	0.005
Baseline age	−0.05	0.03	0.10	0.01	0.25	0.98
Education	0.03	0.05	0.61	0.01	0.05	0.86
Socioeconomic position Medium	0.01	0.21	0.78	−0.21	0.25	0.40
High	0.04	0.24	0.98	−0.20	0.25	0.42
Female	0.14	0.22	0.51	−0.10	0.22	0.67
Dementia cases	−2.31	0.25	<0.001	−1.97	0.28	<0.001
Quadratic slope (acceleration)	−0.12	0.02	<0.001	n/a	n/a	n/a
Years to death from baseline	−0.01	0.00	<0.001	—	—	—
Baseline age	−0.00	0.00	0.35	—	—	—
Education	0.00	0.01	0.90	—	—	—
Socioeconomic position Medium	0.00	0.01	0.90	—	—	—
High	0.01	0.02	0.98	—	—	—
Female	0.00	0.01	0.99	—	—	—
Dementia cases	−0.14	0.02	<0.001	—	—	—
*Random effects (variances)*	—	—	95% CI	—	—	95% CI
Level of performance	0.49	0.03	0.42–0.57	0.71	0.12	0.70–1.21
Linear rate of decline	5.53	0.23	5.09–5.92	5.03	0.39	5.70–7.23
Acceleration	0.94	0.02	0.89–0.96	—	—	—
Residual	2.90	0.07	2.77–3.03	2.30	0.22	1.91–2.78

a The samples included in these analyses represent the participants in each study with mortality data available during the study period, minimum 2 waves of MMSE and complete data on covariates, after excluding participants with dementia at baseline—reason for the reduction in the number of participants included relative to the full samples.

The results of these analyses suggest that the reference OCTO‐Twin individual, a Swedish man who entered the study aged 83, had an average of 7 years of education and was 6 years from death, showed an annual decline of 1.60 (SE = 0.21) MMSE points per year. The decline accelerated by 0.12 (SE = 0.02) points per year closer to the actual time of death.

In contrast, the reference Newcastle 85+ participant, a British man of 85 years, with an average of 10 years of education, and entering the study at around 2.6 years before death, had a linear rate of decline of 1.08 (SE = 0.23) MMSE points per year.

**Figure 1:**
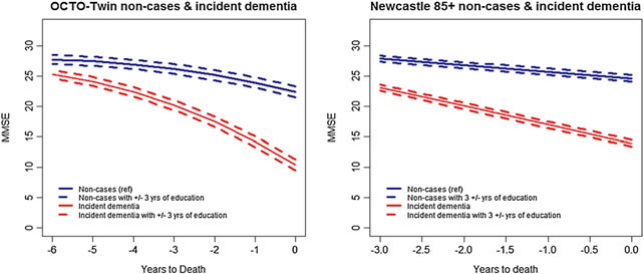
Model estimated mean curves for non‐cases (blue lines) and incident dementia cases (red lines) for different values of education with the additional upper and lower bands of 3 years education (OCTO‐Twin study, left panel and Newcastle 85+, right panel).

Figure 1 illustrates the MMSE trajectories plotted against time to death for non‐cases and dementia cases in each of the two studies for different values of distance to death and education. Additional model estimated mean curves are plotted for the upper and lower bands of 3 years education.

In both studies, those who developed dementia during the study period had significantly lower MMSE scores prior to the time of death compared with those who remained dementia free, with approximately −12.14 (SE = 0.90) and −10.68 (SE = 1.48) points on MMSE in OCTO‐Twin and Newcastle 85+ respectively. Incident dementia cases also experienced a steeper rate of decline with −2.31 (SE = 0.25) in OCTO‐Twin and −1.97 (SE = 0.28) units per year in Newcastle 85+. The acceleration in the rate of terminal decline in OCTO‐Twin study was estimated at −0.14 (SE = 0.02) units per year closer to death.

Although, education was positively associated with the estimated level of MMSE scores before death in the OCTO‐Twin study (0.44 points per year of education, SE = 0.14), it was not found to have an association with the level of performance prior to death in the Newcastle 85+ study. Furthermore, education did not appear to have a significant impact on the rate of change in MMSE in either of the two cohorts investigated.

Higher levels of SEP (medium and high) were not associated with either the level of cognitive performance prior to death or with the rates of terminal decline compared with the low categories in these analyses.

Age at baseline was negatively associated with MMSE scores prior to the time of death in OCTO‐Twin, but not in Newcastle 85+. For each year of age higher than the overall baseline age of 83 years in OCTO‐Twin, the MMSE score was estimated to be lower by −0.49 (SE = 0.10) than the estimated mean score of 24.60 (SE = 0.62). Baseline age was not associated with rate of decline in MMSE in either of these studies. Sex was not associated with either the intercept or the rate of decline in the MMSE in the current examination.

Sensitivity analyses examining the rate of terminal decline within a follow‐up period of only three waves in OCTO‐Twin (to assure similarity to the Newcastle 85 + study) revealed a similar rate of linear change as in Newcastle 85+ (see Supporting Information). The pattern of associations with education, SEP and other covariates remained the same.

## Discussion

In the current study, we examined terminal change in MMSE, and the role of education and SEP on these trajectories, using a coordinated analytical approach in two European population‐based samples of the oldest people from Sweden and the UK. The current investigation addressed how people differ in their final years before death, presenting separate estimates for incident dementia and healthy individuals.

In OCTO‐Twin, where it was possible to investigate polynomial change, the analyses revealed accelerated change prior to death. In Newcastle 85+, which had a shorter period of follow‐up, we found a considerable linear decline. Education was significantly associated with level of performance 6 years before death in one of the two cohorts (OCTO‐Twin), independent of the SEP and other common factors such as age at baseline, sex and time to death at the study entry, but was not associated with the rate of cognitive decline in either of these studies. Furthermore, SEP was not significantly associated with either the level of performance on global cognition prior to death or with the rate of decline within each study.

The current results are consistent with those reported by Wilson, describing terminal decline as a process which starts initially with a slower rate of change, followed by a more pronounced acceleration prior towards death (Wilson *et al.,* 2007). However, the differences across two studies analysed here, in terms of associations between education and level of cognitive performance, as an individual approaches the time to death, re‐emphasise the inconsistency of findings in the literature (Muniz‐Terrera *et al.,* 2011). In the OCTO‐Twin, higher education was predictive of higher global cognitive performance (almost half an MMSE point per year of education) at six years from death, while in the Newcastle 85+, education was not a significant predictor.

The various time metrics used in previous studies (e.g. chronological age, time in study or distance to death) are likely to contribute to the difference in findings related to the acceleration in cognitive decline prior to death.

Another plausible mechanism is that in the absence of neuropathogenic cognitive decline, most people maintain stable or only slightly decline, with a more severe deterioration indicating underlying neurobiological compromise. Periods characterised by slow‐but‐steady cognitive decline may be linked to other abrupt preterminal causes of death (e.g. neoplasms and stroke), which are also recognised to weaken cognitive function and exacerbate cognitive decline (Haj‐Hassan *et al.,* 2014, Lange *et al.,* 2014). These more likely reflect the relatively acute nature of vascular precursors or more specific biological conditions (e.g. organ failure, cancer, dementia, diabetes and respiratory conditions) associated with abrupt effects on cognition and mortality (Hassing *et al.,* 2002, Anstey *et al.,* 2006). Moreover, age is a crucial factor that has been shown to modify the association between the distinctive metabolic risk factors and cognitive decline (Siervo *et al.,* 2014).

In terms of cognitive instrument, MMSE is a screening measure that can be used to systematically assess mental status and identify cognitive impairment, rather than a detailed and thorough measure of global cognition. However, MMSE relies mainly on verbal items (Xu *et al.,* 2002) and lacks a detailed assessment of visuo‐constructive abilities and executive functions (Kirby *et al.,* 2001). Consequently, decline in the MMSE could represent a sensitivity threshold for the verbal task component in the presence of neurodegeneration but could be particularly insensitive to frontal lobe, executive and social function disorders (Hodges, 2011). The maximum score of 30 should be attainable by anyone of average intelligence. However, many patients with early dementia onset could score high on the MMSE, especially if they are younger, have higher education and advanced prior cognitive ability, resulting in a sensitivity reduction of the MMSE scores.

To sum up, the process of terminal decline is a highly complex phenomenon for which we need to take into consideration multiple underlying and intervening factors. The current analyses highlighted an accelerated change in MMSE prior to death in one of the two cohorts investigated (OCTO‐Twin), where the follow‐up period was relatively longer, and a linear decline in the Newcastle 85+ study. More years of education were associated with higher MMSE scores prior to death, but not an attenuation of the rate of change, as reported by other studies. For example, Laukka and colleagues reported that those with more education experienced slower decline in test of Block Design in the proximity of death (Laukka *et al.,* 2006), whilst others who investigated change in MMSE, information, block design and other cognitive domains reported an association of education with performance but not consistent associations with rate of decline (Muniz‐Terrera *et al.,* 2009) (Batterham *et al.,* 2011) (Piccinin *et al.,* 2011).

### Strengths and limitations

A key strength of our study is the use of a coordinated analysis approach, which enables a comparison of results across cohorts. We addressed the same research question, using data from the same cognitive test administered in comparable population‐based samples of 80 + years at baseline, with the same methodological analysis plan, including a similar choice of covariates. Naturally, this type of coordinated analysis approach can provide a far‐reaching foundation by facilitating efficient analysis of cross‐cohort analyses that could help maximise the comparability of results and permit evaluation of study differences (Hofer and Piccinin, 2009, Piccinin and Hofer, 2008). As a novel feature of this investigation, we examined cognitive change within both cohorts in relation to number of years to death, a potentially more sensitive and process‐based parameterisation of time in this age‐group compared with time in study or chronological age which are usually assessed (Sliwinski and Mogle, 2008). However, when moving to a different time metric, the inclusion of time to death from study entry and the baseline age play a similar role by helping to represent selection effects related to age and mortality (Piccinin et al., 2011).

To our knowledge, this is one of the few analyses of terminal decline in the oldest people population, where we were able to separately estimate the expected trajectory of those free of dementia from those diagnosed with dementia during the study period. The results highlight how cognitive decline differs across studies prior to death in two different countries from Europe and that education does not appear to have a neuroprotective role against faster rates of terminal decline, despite a good representation of education range in both cohorts investigated. Lastly, this cross‐cohort investigation provides valuable information regarding the rates of terminal decline in healthy individuals and in those who developed dementia during the study period in two different oldest people non‐institutionalised populations.

We are also aware of some limitations. The terminal decline phenomenon refers usually to an increase in rate of cognitive decline prior to death in individuals free of dementia. However, the British cohort had a shorter period of follow‐up compared with the Swedish cohort, and therefore, we were not able to evaluate whether a change in the rate of decline occurred in both studies. In addition, MMSE is a relatively crude instrument for detecting changes in cognitive function in the oldest people, relying too much on verbal tasks and memory and not enough on other cognitive functions (e.g. speed and executive function), which are known to demonstrate age‐related change. Furthermore, we need to consider the probability of ‘healthy survival’ in longitudinal studies of oldest people and the substantial dropouts in population samples with older people. However, the methodology employed (random effects models) compensated for the missing data aspect. We also faced differences between the two investigated cohorts. The time period examined for the terminal decline was slightly different, with the Swedish cohort having a longer study period to describe an accelerated rate of change, in contrast to the relative shorter period of follow‐up in the British cohort.

Further investigations with more measurement occasions and longer follow‐ups are necessary to better understand the transition from the subtle cognitive changes accompanying age to those of neurological substance. We also need more information about the underlying neurobiological mechanisms that tend to produce accelerated cognitive change prior to death.

## Conclusions

Our results suggest that decline and acceleration of this decline were observable in these studies prior to death. Education was positively associated with MMSE scores in proximity to death, independent of other markers of cognitive reserve such as SEP, in one of the cohorts investigated, but did not appear to be protective against faster rates of terminal decline in the oldest people. As such, our coordinated approach analysis revealed no consistent protection for the cognitive reserve hypothesis (as applied to MMSE), which stipulates a protective role for those with higher education in terms of slower terminal decline.

Key points

- Education protects cognitive performance prior to death, independent of socio-economic position, baseline age and sex in a twin population from Sweden, but not in an older population from the UK.
- Education was not moderating the terminal drop, offering only partial support to cognitive reserve hypothesis. Participants diagnosed with dementia had significantly lower MMSE scores prior to death and experienced twice as steeper terminal decline than those free of dementia, in both of these studies.

## Supporting information

Supporting info item
