# Supplementary material for: The role of cognitive reserve on terminal decline: a cross‐cohort analysis from two European studies: OCTO‐Twin, Sweden, and Newcastle 85+, UK
Source: Int J Geriatr Psychiatry. 2015 Oct 15;31(6):601–10. doi: 10.1002/gps.4366 (PMC4833688; doi:10.1002/gps.4366)
Supplement: Supplementary file 1 — Supporting info item [file GPS-31-601-s001.doc]

# Extra material online:

## *Socioeconomic position*

In the OCTO-twin study, the question asked was: “What has been your main occupation for most of your working life (during the longest period)?” and the responses were coded into low (manual occupations), medium (non-manual occupations) and high (intermediate and professional occupations) according to the Swedish Criteria Group for Occupational Standards (SCG) of the Swedish National Institute for Working Life (NIWL). In the Newcastle 85+, SEP was assessed by main working life occupation, classifying participants through the National Statistics Socio-economic Classification system (NS-SEC) into three categories low, medium and high SEP, equivalent to routine and manual, intermediate and higher managerial, administrative and professional occupations.

## *Missing data patterns in relation to MMSE and education*

We conducted a sensitivity analyses to compare the variation in education for those with missing data in each study (by deriving an indicator for the number of waves missing see Table e1- for OCTO-Twin and Table e2 for Newcastle 85+). We assessed the relationship between the pattern of missingness and the number of years of education in each study, using a Spearman's rank-order correlation. There was no correlation found in either study (r(822) = .06, p =.079 for Newcastle 85+ and r(652) = .057, p = .145 for OCTO-Twin). Given the pattern of these results, we could assume that missingness was not related to education level

Table e1. Mean and standard deviation for education by level of missingness (waves without MMSE data) in OCTO-Twin study

|  | OCTO-Twin study (5 Waves) | |
| --- | --- | --- |
| Missing waves using MMSE | Total | Education years |
|  | N | Mean (SD) |
| All 5 waves missing | 1 | 7.00 |
| Any 4 waves missing | 115 | 7.00 (2.43) |
| Any 3 waves missing | 127 | 7.11 (2.25) |
| Any 2 waves missing | 108 | 7.31 (2.58) |
| Any one wave missing | 87 | 7.12 (1.98) |
| None missing | 217 | 7.14 (2.20) |
| Total | 655[[1]](#footnote-2) | 7.22 (2.35) |

Table e2. Mean and standard deviation for education by level of missingness (waves without MMSE data) in Newcastle 85+ study

|  | Newcastle 85+ study (3 waves) | |
| --- | --- | --- |
| Missing waves using MMSE | Total | Education |
|  | N |  |
| All 3 waves missing | 5 | 9.00 |
| Any two waves missing | 347 | 9.71 (1.67) |
| Any one wave missing | 154 | 10.03 (1.90) |
| None missing | 323 | 10.08 (2.02) |
| Total | 829[[2]](#footnote-3) | 9.13 (1.86) |

## *Cross country comparison*

In order to make a direct comparison between studies, additional analyses were conducted to investigate terminal decline, in which we truncated the study period to only 3 waves of follow-up in OCTO-Twin study to assure a similarity in terms of study design follow-up to the Newcastle 85 + study. The results of these analyses, in which we examined the rate of terminal decline in OCTO-Twin study, within a shorter period of follow-up of only 3 waves, revealed a similar rate of linear change as in the Newcastle 85+ study (see online material). These additional results suggest that the reference participant who participated in OCTO-Twin study, a Swedish man, with an average of 7 years of education and entered the study at around 3.5 years from death, experienced a terminal decline by -0.88 (SE = 0.08), which is relatively slower compared to the linear slope estimated for the reference participant in the British study (Newcastle 85+) of -1.08 (SE = 0.23). However the level of performance on global cognition prior to death was very similar in both studies with 24.29 (SE = 0.62) in the British study and 24.60 (SE = 0.62) in the Swedish cohort.

Table e3. Mean, Standard Error of the Estimates of the Effect of Risk Factors on Random Effects of Terminal Decline Mixed Model for MMSE within each study (with a shorter period of follow-up in OCTO-Twin study truncated to 3 waves to ensure comparison with the Newcastle 85+)

|  | **MMSE in OCTO-Twin study** | | | | | | **MMSE in Newcastle 85+ study** | | | |
| --- | --- | --- | --- | --- | --- | --- | --- | --- | --- | --- |
|  | ***Coef.*** | | ***SE*** | | | ***P value*** | ***Coef.*** | ***SE*** | ***P value*** | |
| ***Fixed Effects*** | |  | |  | **n=552** | |  |  | | **n=352** |
| **Intercept (Level of performance on MMSE prior to death)** | 24.29 | | | 0.62 | **<0.001** | | 24.60 | 0.62 | | **<0.001** |
| **Years to death from baseline** | -0.18 | | | 0.09 | **0.04** | | 0.10 | 0.23 | | 0.67 |
| **Baseline age** | -0.35 | | | 0.10 | **<0.001** | | -0.13 | 0.83 | | 0.88 |
| **Education** | 0.38 | | | 0.13 | **0.004** | | 0.19 | 0.23 | | 0.44 |
| **Socioeconomic position- Medium** | 0.91 | | | 0.69 | 0.14 | | 0.80 | 1.15 | | 0.49 |
| **High** | 1.38 | | | 0.94 | 0.18 | | 1.43 | 0.92 | | 0.12 |
| **Female** | 0.72 | | | 0.66 | 0.27 | | -0.48 | 0.79 | | 0.55 |
| **Dementia cases** | -9.13 | | | 0.89 | **<0.001** | | -10.68 | 1.48 | | **<0.001** |
| **Linear slope (rate of decline)** | -0.88 | | | 0.08 | **<0.001** | | -1.08 | 0.23 | | **<0.001** |
| **Years to death from baseline** | -0.09 | | | 0.01 | **<0.001** | | -0.26 | 0.09 | | **0.005** |
| **Baseline age** | 0.01 | | | 0.01 | 0.96 | | 0.01 | 0.25 | | 0.98 |
| **Education** | 0.01 | | | 0.02 | 0.59 | | 0.01 | 0.05 | | 0.86 |
| **Socioeconomic position -Medium** | 0.04 | | | 0.10 | 0.40 | | -0.21 | 0.25 | | 0.40 |
| **High** | 0.21 | | | 0.13 | 0.67 | | -0.20 | 0.25 | | 0.42 |
| **Female** | 0.05 | | | 0.09 | 0.61 | | -0.10 | 0.22 | | 0.67 |
| **Dementia cases** | -0.85 | | | 0.12 | **<0.001** | | -1.97 | 0.28 | | **<0.001** |
| ***Random Effects (variances)*** | | - | | - | **95% CI** | | - | - | | **95% CI** |
| **Level of performance** | | 0.49 | | 0.03 | 0.42-0.57 | | 0.71 | 0.12 | | 0.70-1.21 |
| **Linear rate of decline** | | 5.53 | | 0.23 | 5.09-5.92 | | 5.03 | 0.39 | | 5.70-7.23 |
| **Residual** | | 2.90 | | 0.07 | 2.77-3.03 | | 2.30 | 0.22 | | 1.91-2.78 |

**Figure e1 Model estimated mean curves for non-cases (blue lines) and incident dementia cases (red lines) in analyses with a shorter period of follow-up in OCTO-Twin study (left panel), truncated to 3 waves to ensure comparison with the Newcastle 85+** (**right panel)** **with the additional upper and lower bands of 3 years education**


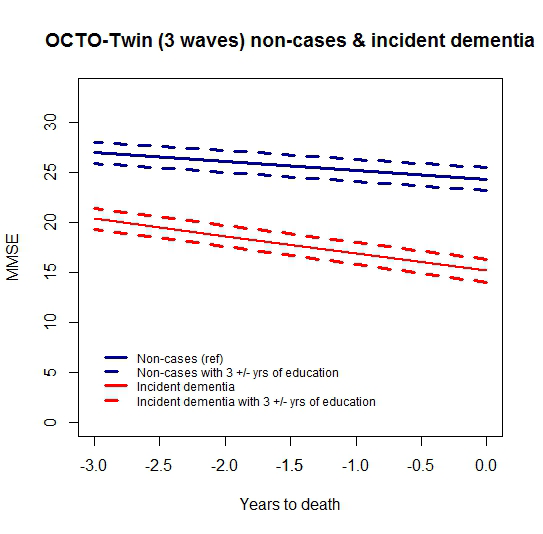

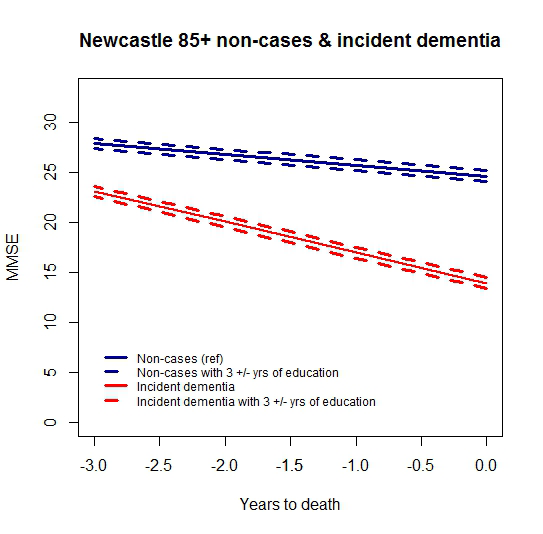


## *Mplus input file for a tobit regression model*

**DATA: file is OCTOTWINterminalcognition.dat;**

**VARIABLE[[3]](#footnote-4):**

**NAMES ARE id mmse1 mmse2 mmse3 mmse4 mmse5 timede1 timede2 timede3**

**timede4 timede5 Educyrs sepmed sephigh gender age1 TD1 DemEver** **DemAge;**

**USEVAR ARE** **mmse1 mmse2 mmse3 mmse4 mmse5 timede1 timede2 timede3**

**timede4 timede5 EduC sepmed sephigh gender age1C TD1C DemEver;**

**USEOBSERVATIONS ARE (DemAge <= age1);**

**TSCORES= timede1 timede2 timede3 timede4 timede5;**

**MISSING ARE ALL (-9999);**

**CENSORED are mmse1-mmse5(A);**

**Define:**

**TD1=deadtime1;**

**TD1C=TD1-(-6);**

**EduC=Educyrs-7;**

**age1C=age1-83;**

**ANALYSIS: type=random;**

**Processors=3;**

**MODEL: i s q | mmse1-mmse5 AT timede1-** **timede5;**

**i s q ON EduC gender TD1C age1C sepmed sephigh DemEver;**

**mmse1(1);**

**mmse2(1);**

**mmse3(1);**

**mmse4(1);**

**mmse5(1);**

**q@0;**

**OUTPUT: sampstat;**

1. In OCTO-Twin study only 655 of the total sample of 702 participants had data for both MMSE and education. The sample of N=352 included in the main analyses of this paper (Table 3) represents the number of participants with data on mortality, MMSE (minimum 2 waves), education, age, socioeconomic position etc. [↑](#footnote-ref-2)
2. In Newcastle 85+ study, 829 of the total sample of 845 participants had data for both MMSE and education. The sample of N=522 included in the main analyses of this paper (Table 3) represents the number of participants with data on mortality, MMSE (minimum 2 waves), education, age, socioeconomic position etc. [↑](#footnote-ref-3)
3. Explanation of the variables: “timede1-5”- Time to death (years) calculated in each study as (Date of death- Date of wave x)/ 365 days; TD1- time to death 1 calculated in each study as (Date of death- Date of wave 1)/ 365 days and included as a covariate in these analyses; Educyrs- years of education; sepmed & sephigh- socioeconomic position medium and high levels included as two separate dummy variables; age1- age at baseline; DemEver (0 they never developed dementia, 1 they developed dementia during the study period; DemAge- age at the time of dementia diagnosis (used for exclusion criteria of those with dementia at baseline(DemAge <= age1)); [↑](#footnote-ref-4)
